# Jasmonates Alleviate Abiotic Stress and Enhance Fruit Quality in Crop Plants: An Updated Review

**DOI:** 10.3390/plants15060975

**Published:** 2026-03-21

**Authors:** María Emma García-Pastor, Alex Erazo-Lara, Pedro Antonio Padilla-González, Domingo Martínez-Romero, María Serrano, Daniel Valero, Vicente Agulló

**Affiliations:** 1 Department of Applied Biology, EPSO-CIAGRO, University Miguel Hernández, Ctra. Beniel km. 3.2, 03312 Orihuela, Alicante, Spain; m.garciap@umh.es (M.E.G.-P.); m.serrano@umh.es (M.S.); 2 Escuela Politécnica Superior de Chimborazo (ESPOCH), Sede Morona Santiago, Macas 140101, Ecuador; alex.erazol@espoch.edu.ec; 3 Department of Food Technology, EPSO-CIAGRO, University Miguel Hernández, Ctra. Beniel km. 3.2, 03312 Orihuela, Alicante, Spain; ppadilla@umh.es (P.A.P.-G.); dmromero@umh.es (D.M.-R.)

**Keywords:** crosstalk, jasmonic acid, plant hormones, postharvest, preharvest, ripening, yield

## Abstract

Jasmonic acid (JA) and its derivative, methyl jasmonate (MeJa), are naturally occurring plant hormones involved in alleviating abiotic stresses, such as exposure to extreme temperatures (cold or heat), flooding and drought. JA content increased following MeJa applications at pre- or postharvest, regulating several physiological and biochemical processes during fruit growth and ripening. As a preharvest treatment, MeJa increased crop yield and improved the organoleptic quality of the fruit. Regarding postharvest applications, MeJa reduced the chilling injury symptoms in sensitive fruits when they were stored at cold temperatures. In addition, there is some evidence of crosstalk between JA and other plant hormones. In this review, we highlight the mechanisms by which jasmonates contribute to plant stress resistance, regulating the biosynthesis and metabolism of abiotic stress and improving fruit quality.

## 1. Introduction

Over the last few decades, extensive research has focused on elucidating the biosynthesis, regulation, and physiological roles of jasmonic acid (JA) and its derivatives. These studies have demonstrated that JA accumulation must be tightly controlled, as a minimum threshold is required to activate its signaling functions, whereas excessive levels may overstimulate defence responses and negatively affect plant growth and development [1]. Thus, jasmonate homeostasis is essential for balancing stress adaptation and growth-related processes.

Jasmonic acid (JA), along with its derivatives methyl jasmonate (MeJa) and jasmonoyl-isoleucine (JA-Ile), is ubiquitously distributed throughout the plant kingdom and is classified as a plant growth regulator (PGR). Jasmonate biosynthesis originates from fatty acid metabolism and was initially identified in the flowers of jasmine (*Jasminum grandiflorum* L.), and later in the culture exudates of the fungus *Lasiodiplodia theobromae*, highlighting its conserved biological relevance across different organisms [2,3]. Subsequent research confirmed that jasmonates act as key signaling molecules involved in the regulation of diverse physiological processes in plants [1].

At the molecular level, jasmonates function within complex signaling networks that integrate developmental cues with environmental signals. The JA signaling pathway is regulated at multiple stages, including biosynthesis, conjugation to bioactive forms, perception, and transcriptional control, enabling plants to modulate the intensity and duration of responses to external stressors [1]. A critical feature of this pathway is its role in coordinating growth–defence trade-offs, as sustained JA signaling may prioritize defence mechanisms over biomass accumulation and yield [1].

Consistent with this regulatory role, JA has been widely associated with enhanced tolerance to abiotic stresses such as drought, salinity, flooding, heavy metal toxicity, and extreme temperatures [4,5,6]. Exogenous application of MeJa has been shown to reinforce antioxidant systems, stimulate secondary metabolism, and improve photosynthetic performance under stress conditions [5]. In horticultural crops, these responses are of particular interest due to their implications for fruit development, postharvest performance, and quality attributes. Moreover, jasmonate-mediated stress responses are closely interconnected with other hormonal pathways, contributing to coordinated physiological adjustments under complex and fluctuating environmental conditions [4,5].

This review highlights the mechanisms by which JA contributes to plant stress resistance and the regulation of biosynthesis and action in stress tolerance, as well as its effect on improving fruit quality.

## 2. Biosynthesis and Catabolism of Jasmonic Acid (JA)

**Figure 1:**
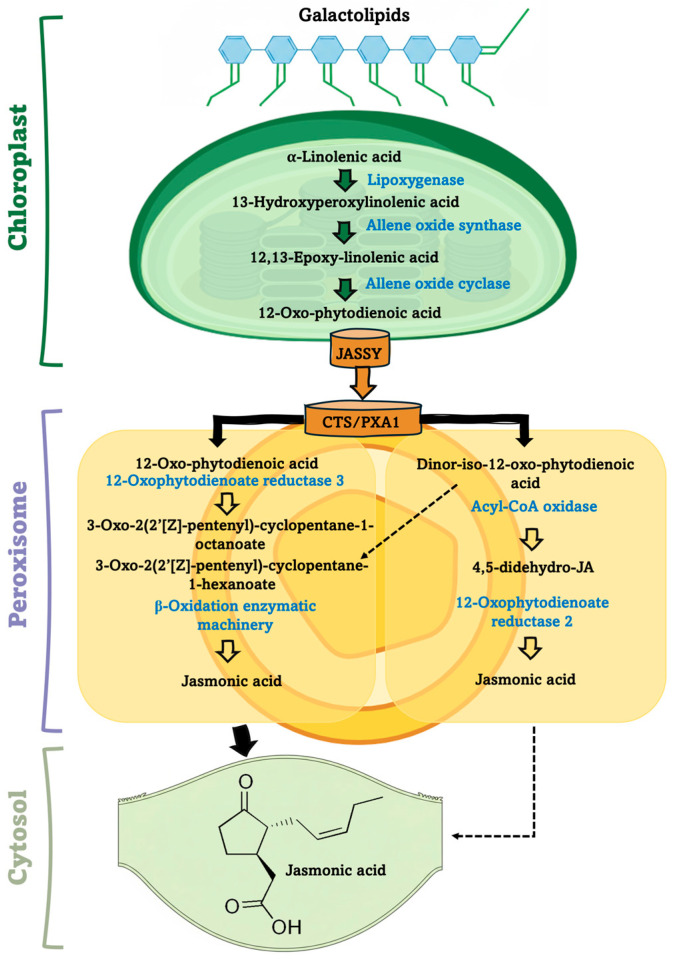
The simplified representation of biosynthetic pathway of jasmonic acid. Abbreviations: JASSY, plastidial 12-OPDA transporter; CTS/PXA1, peroxisomal ATP-binding cassette transporter (also known as COMATOSE or Peroxisomal ABC transporter 1).

The precursor of JA is the fatty acid α-linolenic acid (18:3), which is formed from linoleic acid (18:2) via a desaturation reaction catalysed by the fatty acid desaturase (FAD) enzyme [1]. Free α-linolenic acid is released from chloroplast membrane galactolipids and is oxidized to 13-hydroxyperoxylinolenic acid through a reaction catalyzed by the lipoxygenase enzyme (Figure 1).

The next step is the formation of 12,13-epoxy-linolenic acid through the action of the allene oxide synthase enzyme. This is then converted into 12-oxo-phytodienoic acid (OPDA) through the action of the allene oxide cyclase enzyme. This cyclopentenone intermediate is then transported into the peroxisome by the COMATOSE (CTS) ATP-binding cassette transporter, where, with the help of the 12-oxophytodienoate reductase enzyme, it is converted into 3-oxo-2-(2′-[Z]-pentenyl)-cyclopentane-1-octanoate. This compound undergoes three successive cycles of peroxisomal β-oxidation, catalyzed by the β-oxidation enzymatic machinery, ultimately leading to the formation of JA. In addition to the canonical OPDA-dependent pathway, an alternative route of JA biosynthesis mediated by PXA1 has been described. In this pathway, PXA1 facilitates the direct import into the peroxisome of OPDA-derived acyl-CoA esters or shortened intermediates generated outside the peroxisome. Finally, JA is released into the cytosol to exert its physiological effects (Figure 1).

## 3. Biosynthesis of Jasmonate Derivatives in Plants

**Figure 2:**
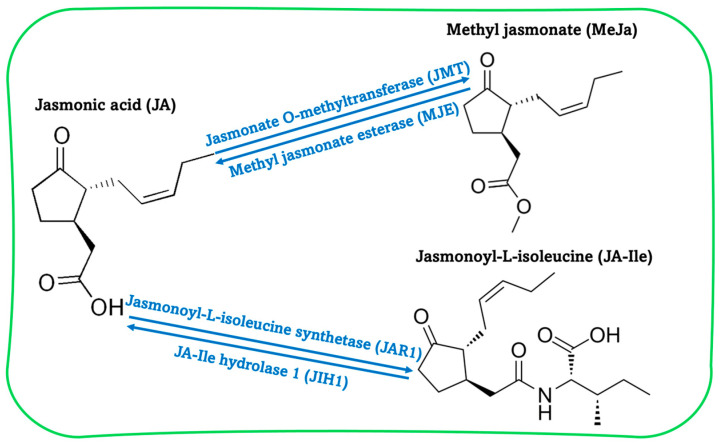
Biosynthetic pathways of methyl jasmonate (MeJa) and jasmonoyl-L-isoleucine (JA-Ile) from jasmonic acid (JA).

Since the discovery of JA in plants in the early 1980s, a large body of research has suggested that a JA derivative, methyl jasmonate (MeJa), is synthesized by the enzyme jasmonate *O*-methyltransferase (JMT) (EC 2.1.1.141). MeJa is a highly volatile, physiologically active molecule found in several food crops, particularly fruits. Furthermore, MeJa is the most active JA derivative used for pre- and postharvest applications [7,8,9]. Another JA derivative is jasmonoyl-L-isoleucine (JA-Ile), formed by the enzyme jasmonoyl-L-isoleucine synthetase (JAR1, or jasmonate-resistant 1) (Figure 2).

## 4. The Role of Jasmonates in Fruit Growth, Ripening and Quality

Diverse mitigation strategies are used to improve fruit quality in crop plants. Phytohormones, like plant growth regulators such as jasmonic acid, were used to enhance fruit quality and abiotic stress tolerance [10]. In fruit crops, ripening is defined as the process that occurs at the end of growth and development, involving physical and chemical changes that lead to fruit of an optimal quality for consumption. Jasmonates (Jas) have been reported to occur at high concentrations in over 160 genera, including algae, fungi, and higher plants [3,11]. JA and MeJa are increasingly abundant in several plant organs, including seeds, leaves and flowers. The highest concentrations are found in fruits, where they play pivotal roles in development, ripening and anthocyanin or carotenoid accumulation [12,13]. Endogenous JA concentrations generally increase greatly at the initial stages of fruit development, then diminish as the fruit matures, with the lowest levels being found at full ripening. This suggests that JA is essential for initiating the ripening process [7]. Notably, both JA and MeJa are considered safe for human consumption. For this reason, they could be used as pre- or postharvest treatments without any detrimental effects on humans [12].

### 4.1. Fruit Growth and Development

It has been observed that plants subjected to abiotic stress increase their concentration of JA, mainly due to their adaptation to these adverse conditions by triggering defence systems in the plant. Additionally, the endogenous JA concentration affects the regulation of fruit growth, development, and ripening. However, in the absence of abiotic stress, JA content depends on the plant species and, as shown in Table 1, even on the studied cultivar. It should be noted that direct comparisons of JA concentrations between all studies in Table 1 are challenging due to differences in reporting units (fresh vs. dry weight).

### 4.2. Crop Yield and Fruit Quality

In recent years, several investigations have been carried out to determine appropriate applications for naturally occurring compounds that can enhance fruit quality at harvest time and during postharvest storage. This is mainly due to consumer concerns about the use of chemical preservatives and legal restrictions. In this context, MeJa plays a pivotal role in providing induced defence against abiotic stress and is a promising candidate [17]. However, the effects of pre-harvest MeJa application on fruit crop yield are not well-known. Table 2 provides examples of the effect of MeJa on the yield of various fruits and vegetables at harvest time.

In general, MeJa treatments led to a higher yield (kg per tree and number of fruits per tree), as occurred with pomegranate (*Punica granatum* L.), pitahaya (*Hylocereus undatus*) and artichoke (*Cynara scolymus* L.). However, in the case of lemons (*Citrus limon* L. Burm. f.), it had no significant effect, probably due to the low concentrations used. Regarding fruit weight, MeJa treatment increased the mass of pitahaya and artichoke fruit, while having no effect on pomegranate and lemon fruit. Accordingly, preharvest application of MeJa at concentrations of 0.1 and 1 mM was found to accelerate the ripening process in table grapes (*Vitis vinifera* L., ‘Magenta’ and ‘Crimson’ cultivars) by promoting berry growth and anthocyanin production [22]. Similarly, in green ‘Lamuyo’ bell pepper (*Capsicum annuum* L.), over two consecutive seasons (2020 and 2021), MeJa foliar sprays at 0.1 and 1.0 mM led to a positive impact on accumulated crop yield, since control plants produced 4.42 ± 0.07 kg per plant, whereas MeJa-treated plants produced significantly higher yields of 5.20 ± 0.08 and 4.89 ± 0.09 kg per plant at 0.1 and 1.0 mM, respectively. Interestingly, the lowest concentration (0.1 mM) produced the best results, suggesting that this dose could be an effective means of increasing the yield of green peppers grown in greenhouses [23]. In two plum (*Prunus domestica* L.) cultivars (‘Black Splendor’ and ‘Royal Rosa’), trees treated with 0.5-, 1- or 2-mM MeJa enhanced fruit size, yield and weight. However, the number of fruits per tree remained unaffected, and the optimal dose for increasing crop yield depended on the cultivar [24]. Conversely, MeJa-treated lemon trees at 0.1-, 0.5- and 1.0-mM did not show any effect on fruit yield [22]. When positive effects were observed, MeJa application could reduce fruit abscission during on-tree growth and ripening and increase the availability of photoassimilates for translocation to the fruit due to higher photosynthesis rates and total chlorophyll concentrations [25]. However, the effects of jasmonates on photosynthesis are not universally positive and appear to be strongly dependent on species, developmental stage, stress conditions and hormone concentration. Several studies have reported that exogenous MeJa or enhanced JA signaling can suppress photosynthetic activity, partly through downregulation of genes encoding key components of the photosynthetic machinery and carbon assimilation pathways [26,27,28]. For instance, in wheat (*Triticum aestivum* L.) exposed to excessive light, both exogenous MeJa and increased endogenous JA levels were shown to compromise tolerance to photoinhibition and the overall capacity of the plants to adapt to this specific stress [29]. Similarly, in cotton (*Gossypium hirsutum* L.) plants attacked by bollworm larvae, activation of JA-mediated defence responses was accompanied by a progressive decline in net CO_2_ assimilation and photoinhibition of both photosystem I and II, reflecting a resource reallocation from photosynthetic processes toward defence metabolism [30]. Moreover, jasmonates are widely recognized as promoters of leaf senescence, and sustained JA signaling has been linked to chlorophyll degradation and reduced photosynthetic capacity. Therefore, the impact of jasmonates on the photosynthetic apparatus should be interpreted within the framework of growth–defence coordination rather than as a uniformly protective effect.

During fruit development, the levels of organic acids are generally inversely correlated with sugar concentrations. Indeed, during maturation and ripening, sugars tend to accumulate due to import or starch degradation, whereas the organic acids that accumulated in young fruits decrease significantly [31]. Malic and citric acids are recognized as the most prevalent organic acids in ripe fruits. In climacteric fruits (such as apples (*Malus domestica* Borkh.), pears (*Pyrus communis* L.), plums, mangoes (*Mangifera indica* L.) and bananas (*Musa acuminata*)), malic acid is used as a specific respiratory substrate, whereas in non-climacteric fruits (such as sweet cherries (*Prunus avium* L.), pomegranates, table grapes, oranges (*Citrus sinensis* L. Osbeck) and lemons), this organic acid continuously accumulates during maturation and ripening. Similarly, citric acid levels decrease during the ripening of climacteric fruits, as it is also used as a respiratory compound. In contrast, there is a progressive accumulation of titratable acidity (TA) in non-climacteric fruits [32]. Sugars accumulate during fruit growth and ripening due to carbon import in the form of sucrose from photosynthetic leaves, which leads to an increase in total soluble solids (TSS). Therefore, the concentrations of sugars and organic acids are essential factors in determining the taste of ripe fleshy fruit, alongside aroma compounds, all of which contribute to the organoleptic quality. Indeed, during ripening, fruits undergo significant changes in terms of firmness, flavor, color, aroma and nutritional composition [33].

**Figure 3:**
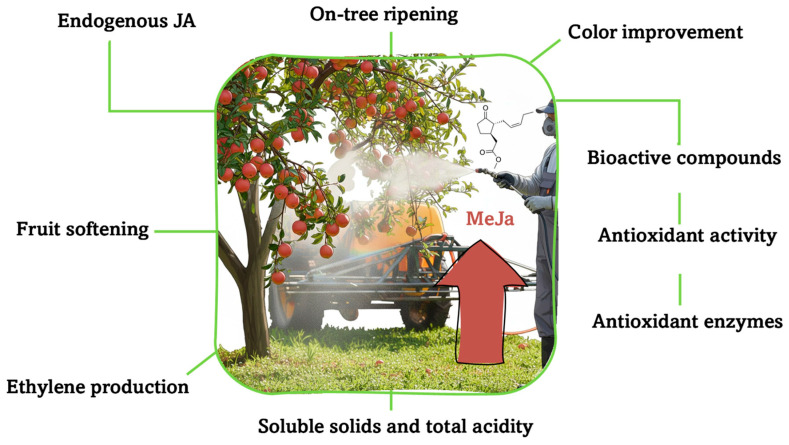
Exogenous methyl jasmonate (MeJa) preharvest application and its physiological effects on on-tree fruit ripening and quality at harvest. The red arrow indicates significant upregulation of the physiological and biochemical parameters shown, mediated by exogenous MeJa application.

Interestingly, the US Food and Drug Administration (FDA) has classified MeJa as a ‘generally recognized as safe’ (GRAS) substance [34]. Accordingly, MeJa could be considered an alternative tool for improving the quality and health-promoting properties of fruits and vegetables [35]. The application of MeJa to fruit has been shown to enhance several aspects of fruit ripening, including increasing endogenous JA and bioactive compounds; improving antioxidant activity; and enhancing color, TSS, and TA (Figure 3).

Preharvest treatment with MeJa influences fruit skin color by promoting chlorophyll degradation and stimulating the biosynthesis of carotenoids or anthocyanins, depending on the species, as well as increasing the accumulation of other phenolic compounds and the antioxidant capacity of the fruit at the time of harvest. In climacteric fruits, JA application accelerates ripening by activating ACC oxidase and the subsequent ethylene emission process, generating volatile aroma compounds [35]. In non-climacteric fruits such as sweet cherries, strawberries (*Fragaria × ananassa*), pomegranates and raspberries (*Rubus idaeus* L.), MeJa treatment enhances the ripening index (RI, or the ratio of TSS to TA), as well as increasing sucrose, fructose and glucose levels [36]. In ‘Monastrell’ wine grapes, MeJa applied at veraison delayed ripening but enhanced anthocyanin accumulation in both berries and wine [37]. Several studies have shown that treating vineyards with MeJa leads to an increase in phenolic compounds (mainly anthocyanins, stilbenes and flavonols) in both grapes and wine [38]. With respect to color, JA plays a pivotal role in anthocyanin biosynthesis, and MeJa increases the expression of genes responsible for anthocyanin accumulation, such as *MdMYB9* and *MdMYB11* [39].

In the ‘Kinnow’ mandarin (*Citrus nobilis* × *Citrus deliciosa*), preharvest MeJa (at concentrations of 0.1, 0.3, 0.5 and 0.7 mM) has been shown to increase fruit firmness and decrease the TSS/TA ratio. The most effective concentration for delaying on-tree ripening was found to be 0.5 mM [40]. In sweet cherry cultivars (‘Ziraat’, ‘Regina’ and ‘Sweetheart’), preharvest MeJa treatment at 2240 mg L^−1^ retarded color changes and decreased TSS, TA and total phenolic content, as well as antioxidant activity. However, a delay in harvest time increased the bioactive compounds [41]. In peach (*Prunus persica* L. Batsch) fruits, MeJa at concentrations of 10 and 100 mM, when applied in the field, stimulated peel chlorophyll degradation and concurrent anthocyanin biosynthesis, as well as enhancing the antioxidant enzymes CAT, POD, SOD and PPO. It also delayed fruit softening by inhibiting the cell wall enzymes PG and cellulase, which has been attributed to increased ethylene production [42]. Preharvest treatment of lemon trees with MeJa (at 0.1, 0.5 and 1 mM) increased TA, firmness and total phenolic content in both flavedo and juice tissues, as well as antioxidant enzymes (CAT, POD and APX) in the flavedo, without affecting external color [20]. Similarly, preharvest MeJa treatments of pomegranate trees at 1 and 5 mM accelerated on-tree ripening, whereas 10 mM retarded it, as evidenced by increased firmness and improved color of the skin and arils [18]. In lettuce (*Lactuca serriola*), foliar spraying with MeJa (at 200 and 400 μM) increased the activity of the antioxidant enzymes CAT, SOD and APX and enhanced total phenolic compounds; however, total flavonoids did not change significantly [43]. Overall, preharvest MeJa application increases photosynthesis rates and chlorophyll breakdown, thereby improving crop yield and quality at harvest. This is attributed to increased endogenous JA levels, as shown in Figure 3.

## 5. The Role of Jasmonates in Mitigating Abiotic Stress

**Figure 4:**
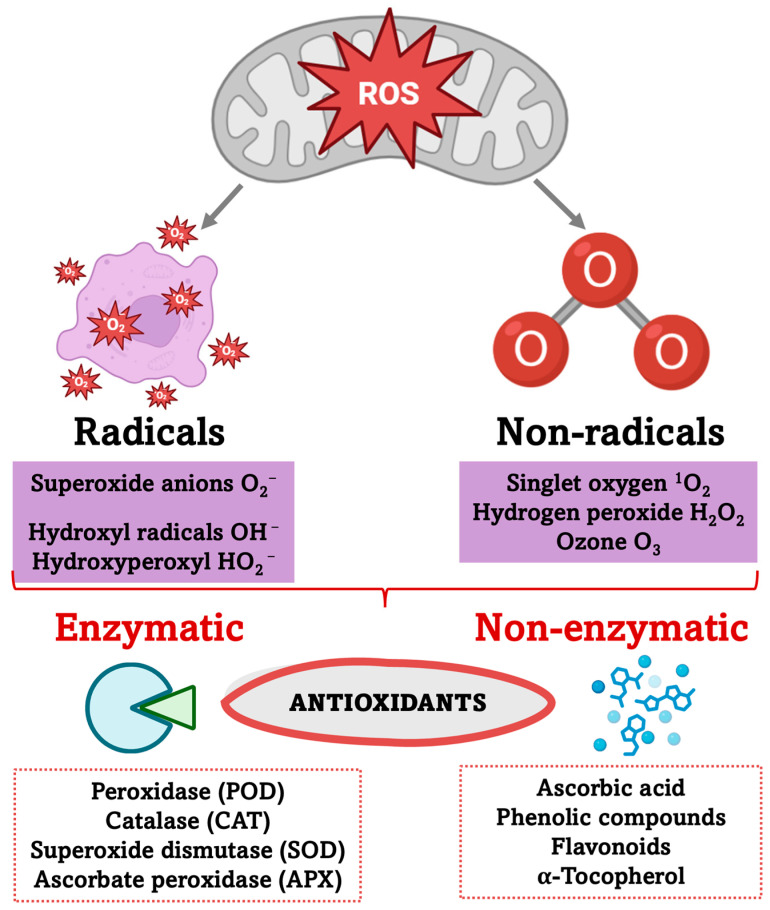
Classification of reactive oxygen species (ROS) and the plant antioxidant defence system.

Globally, climate change is having a negative impact on crop growth and productivity by stimulating various abiotic stresses, including salinity, low and high temperatures, drought, heavy metals and UV radiation. These stressful events trigger an excessive production of reactive oxygen species (ROS) in plant organelles such as mitochondria, chloroplasts and peroxisomes. While ROS plays essential signaling roles in regulating cellular redox status, excessive production disrupts cell integrity, particularly affecting protein denaturation, lipid peroxidation and nucleic acid damage, collectively leading to cell death [44]. ROS are regulated by scavenging through both enzymatic and non-enzymatic antioxidant systems; the enzymatic systems are related to defence mechanisms (Figure 4).

**Figure 5:**
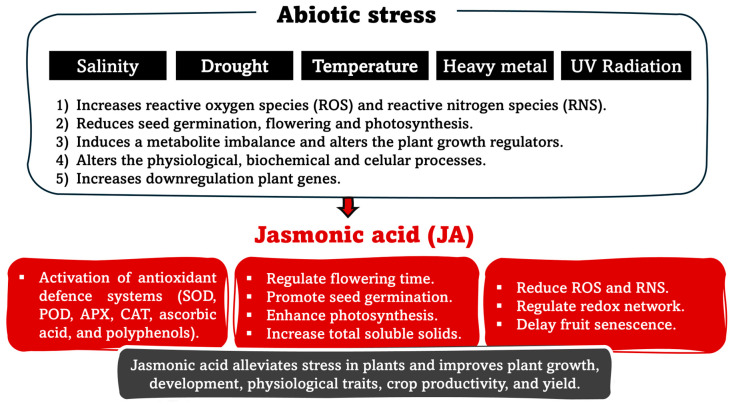
Schematic representation of the role of jasmonic acid (JA) in alleviating abiotic stress-induced physiological and biochemical impairments in plants. Abbreviations: SOD is superoxide dismutase; POD is peroxidase; APX is ascorbate peroxidase; and CAT is catalase.

Among the plant hormones, JA and MeJa have been suggested as signaling pathways that alleviate abiotic stress tolerance and related growth responses. This enables plants to remain healthy under adverse conditions (Figure 5).

JA and MeJa are the most used plant growth regulators for alleviating abiotic stress and pathogenic microbes, as they trigger antioxidant enzymes that quench ROS generation [10,45]. For example, under salinity stress, JA plays a vital role in seed germination since the presence of salts in the soil reduces water uptake by the roots. This increases osmosis, which inhibits seed germination. JA application increases water use efficiency, decreases the presence of osmolytes, and favors seed germination [46]. However, JA has been reported to inhibit seed germination in lupin (*Lupinus albus* L.), in which MeJa, at high concentrations (10^−4^ to 10^−3^ M), suppressed the germination rate, resulting in decreased growth rate of seedlings [47]. Accordingly, in thale cress (*Arabidopsis thaliana*), MeJA triggers the inhibition of etiolation growth, resulting in defects in seedling emergence, which may be attributed to the fact that jasmonate biosynthesis might affect seedling germination [48]. In addition, salt stress induces oxidative damage, affecting the cell membrane and leading to lipid peroxidation and the leakage of several osmolytes. JA treatment decreases accumulated ROS and protects cell membranes from toxic salinity by stabilizing the membrane and decreasing the production of malondialdehyde [49]. Drought is another abiotic stressor that induces adverse effects on plant growth and crop yield. It does this by altering cell membrane integrity, reducing gas exchange due to stomatal closure, and resulting in diminished photosynthetic efficiency [50]. It has been revealed that JA signaling pathways are connected to increased tolerance to drought stress [51]. For example, strawberry plants can withstand drought stress using exogenous JA [52]. An increasing number of studies have shown that JA induces the expression of drought tolerance-related genes, including *IbMYB116* and *GmGA15*, which encode transcription factors involved in the regulation of stress-responsive gene networks [53].

Cold stress is classified as exposure to temperatures (0–15 °C) and freezing temperatures (<0 °C) and is considered a severe environmental event that limits crop productivity. Exposure of plants to cold temperatures induces oxidative stress, altering cell function. In response, antioxidant enzymes are activated to counteract ROS generation, but their activities are sometimes insufficient to induce cold tolerance. For example, apples can withstand freezing temperatures due to the action of JA, which enhances the expression of the *MdCBFF1*, *MdCBFF2* and *MdCBFF3* genes, thereby inducing cold tolerance [5,51]. Similarly, MeJa treatment enhances plant resistance to low temperatures. This treatment triggers the production of genes responsible for JA biosynthesis and increases the endogenous JA concentration, stimulating the synthesis of biochemical compounds (especially polyphenols) and enzymatic antioxidants. Thus, MeJa could be an effective means of mitigating the harmful effects of cold-temperature stress [54].

**Figure 6:**
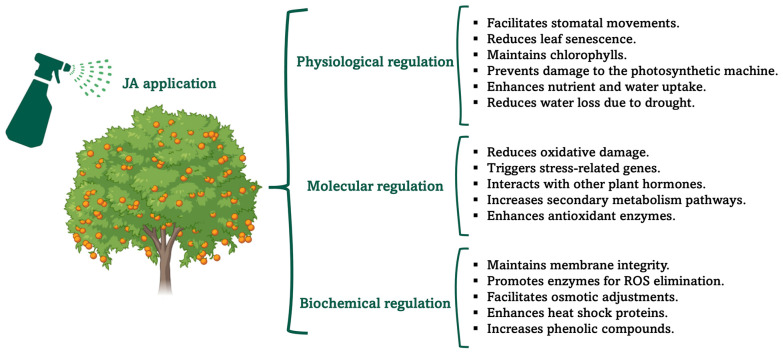
Overview of physiological, molecular, and biochemical regulations mediated by exogenous jasmonic acid (JA) application in fruit trees. Abbreviations: ROS are reactive oxygen species.

High temperatures also alter several physiological and biochemical processes that affect plant growth and development. Elevated temperatures induce cellular membrane damage, reduce the rate of photosynthesis and enhance the rate of respiration, which contributes to a reduction in plant growth, development and yield [55]. Increased levels of JA have been shown to alleviate the effects of heat stress. Exogenous MeJa application enhances JA accumulation, promoting antioxidant defence, osmolyte adjustment and the expression of stress-responsive genes, including heat shock proteins (HSPs), which contribute to thermotolerance under acute heat stress [56,57]. However, the role of jasmonates under elevated temperatures is not uniformly protective. Under moderately warm conditions that induce thermomorphogenesis, warm temperatures enhance the expression of catabolic JA enzymes, leading to reduced levels of bioactive JA-Ile and stabilization of JAZ repressors [58]. This attenuation of JA signaling facilitates elongation growth responses mediated by thermomorphogenic regulators. These findings indicate that sustained JA signaling can restrict adaptive growth responses to warm temperatures and that plants actively downregulate JA signaling to permit thermomorphogenesis. Thus, jasmonates may enhance survival under acute heat stress while negatively regulating growth-related adaptations to elevated ambient temperatures, reflecting a context-dependent growth–defence balance. Available evidence suggests that JA accumulation is induced by ROS production in the presence of stressors, and that JA can induce the formation of antioxidants [6]. Figure 6 shows a summary of the roles of JA in plants in terms of physiological, biochemical and molecular regulation, which reduces the negative impact of stress on plant yield.

## 6. Methyl Jasmonate: Improving Postharvest Fruit Quality and Resilience

**Figure 7:**
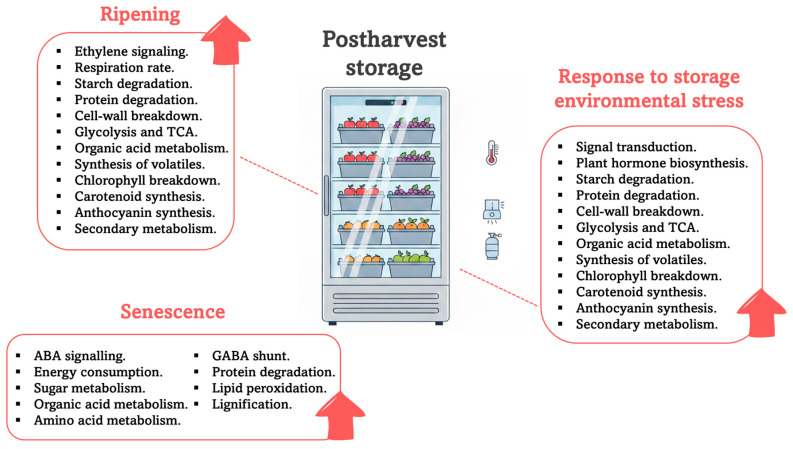
Overview of the regulatory pathways governing ripening, senescence, and stress adaptation during cold storage; red arrows indicate the induction of specific metabolic routes. Abbreviations: TCA is tricarboxylic acid; ABA is abscisic acid; and GABA is gamma-aminobutyric acid.

Fruit quality refers to the physicochemical characteristics that greatly impact consumer acceptance. For most fruits, it is difficult to define a universal standard of quality, mainly due to various factors throughout the horticultural supply chain, including pre- and post-harvest management, as well as marketing and socio-economic factors, which influence consumers’ perceptions of the quality of fruit they purchase [59]. From a pre-harvest perspective, fruit quality can have a significant effect on organoleptic properties, physicochemical properties, and the content of bioactive compounds related to health attributes. All these parameters depend largely on genetic material, fruit development and ripening at harvest, agronomic practices and environmental factors, especially those related to abiotic stress due to climate change [60]. From a postharvest perspective, storability potential and shelf-life are closely related to the preservation of fruit quality, and thus postharvest management, including handling and packaging, affects the maximum storage period and the quality of the fruit [61]. Primary metabolism does not enhance flesh flavor but increases the special components of energy supply due to physiological activities during postharvest fruit storage. Thus, the respiration rate influences primary metabolism during postharvest storage of fruit, affecting processes such as starch degradation, glycolysis, the tricarboxylic acid (TCA) cycle, and the metabolism of lipids, amino acids, and organic acids [62]. These metabolic changes affect the composition and content of sugars, amino acids, organic acids, and fatty acids (Figure 7).

Chilling injury (CI) develops in most fruits when they are stored at temperatures below 10–15 °C, depending on the plant species. This physiological disorder poses a challenge due to the reduced storage life of 7–40 days on average. Symptoms of CI include discoloration of the peel and flesh, water-soaking, off-flavor, failure to ripen, and accelerated growth of molds, leading to decay. These symptoms are exacerbated when the fruit is transferred from cold rooms to higher temperatures, resulting in abnormal ripening because of CI. The best way to control CI is to avoid exposure to chilling temperatures, although this compromises the shelf-life. The minimum safe storage temperature for selected fruits is shown in Table 3.

A variety of physical and chemical treatments are available to extend the storability and shelf-life of chilling-sensitive fruits and reduce quality losses. These include modified atmosphere packaging (MAP), polyamine (PA) applications, edible coatings and 1-methylcyclopropene (1-MCP), among others [32]. In recent years, plant hormones have emerged as a technology for improving fruit quality and shelf life when applied as pre- or postharvest treatments. In this regard, it has been reported that MeJa activates defence mechanisms against chilling stress by regulating biochemical processes and preventing deterioration of the fruit by decreasing oxidative stress [63]. Exogenous MeJa application increases the biosynthesis of bioactive polyphenol compounds and antioxidant enzymes; improves fruit quality properties, such as color, firmness, TSS and TA; and prevents weight loss during postharvest storage [18,19,20,21,22,23,24,63].

### 6.1. Effects of Preharvest MeJa Application on Postharvest Fruit Quality and Secondary Metabolism

Over the past decade, the use of JA derivatives has been shown to delay fruit ripening and senescence [55]. However, its effect depends on the concentration applied [18,19,20,21,22]. In addition, the application of MeJa in the field reduced CI symptoms during the postharvest storage of pomegranates and improved aril color [18]. Regarding fruit firmness, MeJa was also found to impact this important parameter, with a delay in fruit softening reported in persimmon (*Diospyros kaki* L.f.), peach and apple, although a low effect was exhibited in pitahaya [64]. These results suggest that the effect of MeJa depends on the plant species, cultivar, and concentration used. Moreover, MeJa significantly affects changes in the color of the fruit skin, the content of anthocyanins and phenolic compounds, and the antioxidant capacity of mature fruit crops [3,37]. Applying MeJa to apples three weeks before the commercial harvest revealed an increase in fruit coloration without affecting ethylene production or fruit firmness during storage [65]. Exogenous MeJa treatment at 1.5 mM affected apple carbohydrate metabolism by decreasing the concentration of starch due to enhanced α-amylase and β-amylase activities, leading to the accumulation of reducing sugars such as fructose, glucose and sucrose [66]. Regarding organic acids, preharvest of MeJa at 20 mg L^−1^ increased the content of several sugars in Chinese dwarf cherry (*Prunus humilis*) while reducing the concentration of malic and citric acids. This enhanced flavor-related components such as the RI index and the sugar–acid ratio [67].

Treatment with MeJa can modify the nutritional quality of tomatoes (*Solanum lycopersicum* L.), as application of 100 μM MeJa has been shown to reduce total pectin content and increase the concentration of carotenoids, particularly lycopene, as well as the levels of glucose, fructose, total phenolic compounds and flavonoids in mature fruit [68]. Anthocyanins are water-soluble, natural, pigmented flavonoid compounds that give fruits their red, blue and purple colors. JA application is used to maintain fruit quality during storage and improve fruit color [69,70]. For example, preharvest application of MeJa enhanced the anthocyanin concentration and antioxidant activity of blackcurrants (*Ribes nigrum* L.) [71]. Preharvest application of 1 and 2 mM MeJa to pomegranates reduced CI, enhanced the color of the arils, and increased total phenolics, total anthocyanins, and total antioxidant activity [72].

### 6.2. Effects of Postharvest MeJa Application on Postharvest Fruit Quality and Secondary Metabolism

**Figure 8:**
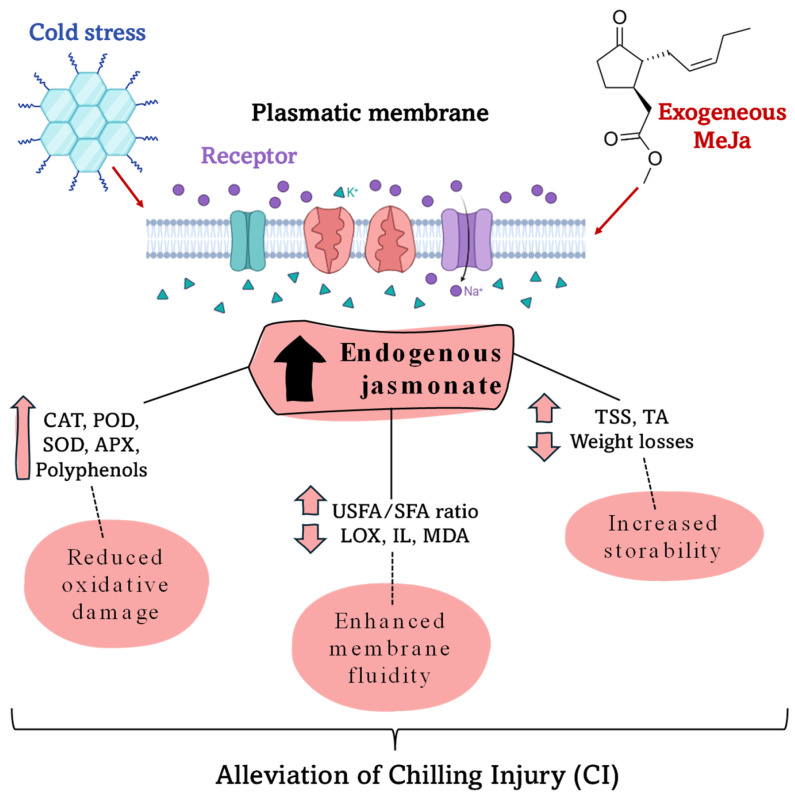
Proposed mechanisms of exogenous methyl jasmonate (MeJa) in alleviating chilling injury (CI) and enhancing cold tolerance in fruits. Abbreviations: CAT is catalase; POD is peroxidase; SOD is superoxide dismutase; APX is ascorbate peroxidase; USFAs are unsaturated fatty acids; SFA are saturated fatty acids; LOX is lipoxygenase; IL is ion leakage; MDA is malondialdehyde; TSS are total soluble solids; and TA is titratable acidity. Na^+^ and K^+^ are corresponded to pomp Na/K for maintaining the structure of the plasmatic membrane.

Postharvest CI induces several physiological factors in fruits, especially alterations to the plasma membrane, energy deficits and oxidative stress [73]. Exposing fruits to cold temperatures induced a significant shift in the accumulation of reactive oxygen species (ROS), primarily superoxide ions (O_2_^•−^) and hydrogen peroxide (H_2_O_2_), resulting in fruit damage. To mitigate these detrimental stresses, plants possess a sophisticated antioxidant system, including either enzymatic and non-enzymatic antioxidants, to prevent cell damage and combat unfavorable environmental stress [74]. Recently, the mechanism by which MeJa reduces CI symptoms has been attributed to increased antioxidant activity and maintenance of mitochondrial stability. Thus, in pear and mango, postharvest MeJa enhances the antioxidant system and suppresses the membrane lipid peroxidation provoked by ROS. This maintains the integrity of the mitochondrial structure and alleviates CI occurrence under chilling stress conditions [75,76]. MeJa has also been shown to mitigate CI in immature fruits such as aubergines (*Solanum melongena* L.), cucumbers (*Cucumis sativus* L.) and courgettes (*Cucurbita pepo* L.) [77]. In addition, postharvest MeJa at concentrations of 0.1, 0.3, 0.5, and 0.7 mM increased the concentrations of total carotenoids, total phenolic compounds, flavonoids and vitamin C in ‘Kinnow’ mandarin, mainly due to the stimulation of enzymes responsible for the corresponding biosynthesis [40]. These results suggest that maintaining proper ROS homeostasis is essential for reducing CI symptoms in fruit, as can be observed in Table 4 and Figure 8.

The receptors are located in the plasma membrane, which is the main target of cold temperature, responsible for transforming the liquid-crystalline structure into a solid gel and losing fluid permeability. The MeJa application induced enhancement of JA endogenous content, which triggered a reduction in oxidative damage, increased membrane fluidity, and increased fruit storability. Accordingly, in pomegranate (very sensitive to CI), it reduced EL and MDA, in addition to increasing the plasmatic membrane instauration (a higher percentage of unsaturated fatty acids (UFAs) with respect to saturated fatty acids (SFAs); without MeJa, CI reduced the USFA/SFA ratio [81,87].

## 7. Jasmonates and Crosstalk with Plant Hormones in Fruit Growth and Ripening

**Figure 9:**
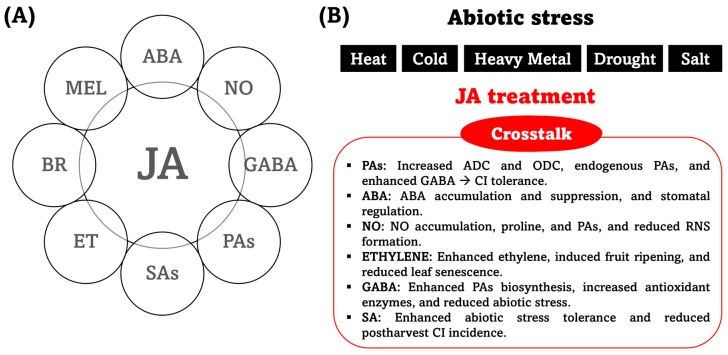
Integration of jasmonic acid (JA) in plant signaling networks; hormonal crosstalk landscape between JA and key signaling molecules (**A**) and physiological and biochemical mechanisms mediated by JA treatment to enhance abiotic stress tolerance (**B**). Abbreviations: ABA is abscisic acid; NO is nitric oxide; GABA is gamma-aminobutyric acid; PAs are polyamines; SAs are salicylates; ET is ethylene; BR are brassinosteroids; MEL is melatonin; ADC is arginine decarboxylase; ODC is ornithine decarboxylase; CI is chilling injury; and RNS are reactive nitrogen species.

Phytohormones do not play an independent role in plant organs; rather, they are involved in several biochemical processes. This suggests clear evidence that MeJa interacts with other plant hormones through crosstalk in response to both biotic and abiotic stresses [88,89,90]. Some reports support the idea of a cross-link between JA and other plant hormones, including abscisic acid (ABA), nitric oxide (NO), gamma-aminobutyric acid (GABA), polyamines (PAs), salicylates (SAs), ethylene (ET), brassinosteroids (BR), and melatonin (MEL), as can be observed in Figure 9A,B.

JA and SAs are both essential phytohormones that play pivotal roles in signaling pathways relating to plant defence against biotic and abiotic stresses. For example, JA plays a key role in defence against necrotrophic pathogens, while SAs are also essential for resistance against biotrophic pathogens. JA and SA also interact with other phytohormones, such as abscisic acid (ABA) and ethylene, forming a complex regulatory network that coordinates stress responses with growth-related processes. This hormonal crosstalk modulates defense activation while preventing excessive resource allocation to defense at the expense of plant development, thereby fine-tuning immune responses against pathogens while maintaining growth homeostasis [91]. This involves increasing antioxidant enzymes, improving disease resistance and promoting the accumulation of molecules such as polyphenol compounds. Both JA and MeJa improve fruit quality by enhancing bioactive compounds and antioxidant activity when applied at either the pre- or postharvest stage. Specifically, they improve skin color by increasing anthocyanins and other phenolic compounds. Additionally, MeJa treatments accelerate fruit ripening in conjunction with ethylene production, affecting parameters related to ripening, cold tolerance, and disease resistance [3]. Although multiple plant hormones contribute to the regulation of fruit growth, development and ripening, ABA and ethylene have been reported as the main regulators of ripening in both non-climacteric and climacteric fruits [65,92]. Given the reported synergistic relationship between ethylene and MeJa in regulating ripening, it is notable that MeJa plays a key role in generating volatile aroma compounds, as observed in mango [16]. In non-climacteric fruits such as strawberries, ABA has been postulated to be the main plant hormone affecting ripening. Thus, during strawberry fruit development and ripening, an increase in endogenous ABA due to MeJa treatment was found to induce red color by enhancing the anthocyanin concentration [93].

The postharvest quality of fruit and the extension of its shelf-life have been reported to depend on the role of NO as a signaling molecule. Thus, crosstalk between JA and NO is involved in maintaining fruit quality during the postharvest period [94]. For example, exogenous MeJa application enhances resistance of blueberries (*Vaccinium corymbosum* L.) to *Botrytis cinerea* infection and stimulates NO biosynthesis, which is also associated with reduced chilling injury symptoms during cold storage [95,96,97]. In addition, in cucumber, which is highly susceptible to CI, postharvest NO application improved tolerance to low temperatures mainly by modulating plant hormone signaling, including JA, ABA and other phytohormones [98]. These authors observed that low temperatures promoted JA biosynthesis from α-linolenic acid by regulating the expression of *LOX2S*, *AOC*, *AOS* and *OPR* genes, with NO participation. There is also information about the interaction between MEL and MeJa during the postharvest period of fruit. It is evident that MEL acts as a crucial signaling compound in a variety of physiological functions in fruits under abiotic stress [99,100]. In addition, MeJa induces the expression of genes responsible for melatonin biosynthesis and inhibits JA biosynthesis, which in turn inhibits MEL biosynthesis [101]. Postharvest application of MEL upregulates endogenous JA content, alleviating CI injury symptoms in a wide range of fruits [102]. Conversely, MEL suppressed the enhanced anthocyanin concentration induced by JA application [103]. Subsequently, MeJa increased endogenous MEL, suggesting that MeJa promotes cold acclimation in plants [104].

There is little literature on the relationship between JA and GABA. In plums, the application of exogenous MeJa inhibits CI occurrence and related symptoms, such as internal browning and EL. The most significant effects were observed at a MeJa concentration of 10 μM. Additionally, MeJa decreases the overproduction of ROS by enhancing antioxidant enzymes such as SOD, CAT, and APX, thereby maintaining mitochondrial membrane integrity. Furthermore, MeJa increased GABA levels by stimulating the activity of GABA transaminase, succinate dehydrogenase, and cytochrome C oxidase, among others. These results suggest that activation of the GABA shunt by MeJa could enhance the energy supply. Thus, the increased cold tolerance of plum fruit after MeJa treatment was partially achieved through activation of the GABA system, the antioxidant system, and the energy supply [105].

The PAs are cationic, low-molecular-weight, nitrogenous compounds that are involved in enhancing cell division and fruit development, as well as delaying senescence, in a wide range of fruit species. Unlike classic phytohormones, they are typically found at higher concentrations in the mM range, despite being considered plant growth regulators and mediators of other plant hormones [106]. However, the relationship between JA and PAs has been poorly studied. The three main PAs found in plants are putrescine (PUT), spermidine (SPD), and spermine (SPM). These play a role in plant growth, fruit ripening, and as an adaptive mechanism in response to abiotic stress [32,107]. A previous study found a positive correlation between JA and PAs in artificially drought-stressed tomato seedlings treated with polyethylene glycol [108]. An increase in PUT concentration enhanced the JA content in both the shoot and the root, suggesting a synergistic effect of these plant hormones in adapting to this abiotic stress. In tomato, MeJa at 0.05 mM alleviated CI by decreasing MDA concentration and increasing the antioxidant activities of SOD, CAT, and POD, as well as PAs and proline accumulation [109]. These results suggest that reduced CI in tomato is partially due to PA metabolism.

The phytohormone ET is the only gaseous plant hormone that acts as a potent regulator of plant defence against biotic stress, primarily in the form of bacterial pathogens, viruses, insects, and necrotrophic fungi [110,111]. However, ET’s most important role is controlling fruit ripening, particularly in climacteric fruits [112,113]. Recent research has studied the role of ET during ripening and senescence in stressed fruits. There is increasing evidence of the relationship between JA and ET, although they exhibit either synergistic or antagonistic effects in the regulation of fruit ripening under abiotic stress conditions. In apples, preharvest treatment with 10 mM MeJa 21 days before harvest enhanced red coloration without compromising storage potential, as there were no significant differences in ET production rates between control and MeJa-treated fruits [65]. A recent report using a JA-deficient mutant of the lipoxygenase gene demonstrated the relationship between ET and JA during the development of flowers of courgettes [114,115]. It is known that JAs activate the biosynthesis of ethylene genes and thus enhance the climacteric peak in ethylene emission rates [116].

In recent years, plant hormones and growth regulators have been considered eco-friendly pre- and postharvest tools for enhancing tolerance to abiotic stress. Among these, BR, a new family of phytohormones derived from sterol precursors, has emerged through signal transduction pathway crosstalk in many crops [117,118]. Exogenously applying BR and JA improves drought stress tolerance by increasing photosynthesis rates and antioxidant and osmolyte levels [119], suggesting that both JA and BR are essential for maintaining plant resilience against drought stress [120]. Generally, the application of BR improves grape flavor and color, although organic acids decrease. However, MeJa treatment enhances total acidity and sugars [121]. These results suggest that BR could inhibit the delay of fruit ripening caused by JA [122].

## 8. Climatic Changes in the Agronomy of Fruit Species

From an agricultural perspective, the changing global environment poses a challenge due to its impact on reducing crop productivity at a time when more food is needed to feed a growing population. According to United Nations predictions, the global population will exceed 9 billion by 2050. This will require a staggering 70% increase in agricultural production to ensure global food security, as reported by Francini and Sebastiani [123]. However, abnormal weather conditions make it difficult to comply with this goal due to the incidence of abiotic stress [124]. These challenges highlight the urgent necessity of finding innovative solutions to fulfil the United Nations’ Sustainable Development Goals (SDGs), particularly SDG 2 (Zero Hunger), SDG 13 (Climate Action) and SDG 12 (Responsible Consumption and Production). SDG 2 and SDG 12 are closely linked, as they both focus on environmental sustainability and resource management [125]. The major consequence of climate change is increased exposure of plants to abiotic stress, which induces crucial physiological changes for adaptation. Fruit crop production must adapt to increased CO_2_ levels, higher temperatures and heavy rainfall, since plant growth and development are sensitive to these changes. Nowadays, climate change is mainly caused by the Industrial Revolution and daily human activities, which maximize the greenhouse effect and increase the Earth’s temperature [126].

## 9. Conclusions

This review provides an update on the mechanisms of action of JA on plant tolerance to abiotic stress, with a particular focus on its impact on improving fruit quality. MeJa treatments led to higher yield, expressed in terms of kg per tree and number of fruits per tree. Preharvest application of MeJa to fruit crops enhances endogenous JA and, in turn, improves quality characteristics such as color, firmness, TSS and TA, as well as enhancing the content of bioactive compounds and related antioxidant activity. Under abiotic stress, increased ROS accumulation was counteracted by MeJa treatment through its effect on physiological, biochemical, and molecular regulators.

Storage at cold temperatures induced CI symptoms, which were alleviated by JA treatment through pre- or postharvest applications, thereby retarding senescence and preserving fruit quality attributes. Attention has been given to the interaction between plant hormones, which has demonstrated that JA does not act alone, but rather in conjunction with different phytohormones. Overall, MeJa application on a commercial scale could be considered an excellent strategy for regulating fruit ripening and promoting health-enhancing antioxidants while maintaining fruit quality. In summary, the JA pathway acts as a pivotal hub in the plant’s hormonal signaling network, integrating inputs from ABA, ET, NO, and others to orchestrate a complex and adaptive response to environmental challenges [127].

## 10. Future Trends

Over the past decade, significant progress has been made in our understanding of the processes that regulate the biosynthesis and metabolism of JA, as well as its signaling pathways. Although there is a wealth of scientific knowledge on the action of JA and its effects on crop yield and fruit quality traits, further research is required. For example, JA signaling and its relationship with other metabolic pathways require further elucidation. Additionally, the role of JA in fungal necrotrophic fruit diseases needs to be explored. Furthermore, little research has been conducted on the interaction between JA and other plant hormones, particularly at the transcriptional and post-translational levels during fruit postharvest storage. Furthermore, additional molecular and biotechnological approaches are required to increase our knowledge of JA physiology in plants for application in horticulture. The putative beneficial effects of JA and MeJa as alternative tools for regulating abiotic stress are of particular interest. From a commercial perspective, comprehensive research into more crop species and cultivars is needed to ensure food safety and promote the sustainability of agriculture.

## Figures and Tables

**Table 1:** Examples of endogenous jasmonic acid (JA) in various fruits at different maturity stages.

Fruit	Cultivar	Maturity Stage	Content (ng g^−1^) ^ϯ^	Reference
Apple	‘Tsugaru’	Immature	18.71 *^,ł^	[13]
		Mature	33.22 ^ł^	
Sweet cherry	‘Satohnishiki’	Immature	10.09 *^,ł^	[13]
		Mature	2.31 ^ł^	
Sweet cherry	‘Prime Giant’	Immature	150 *^,ϒ^	[14]
		Mature	320 ^ϒ^	
Strawberry	‘Benihoppe’	Immature	85 *^,ł^	[15]
		Mature	20 ^ł^	
Mango	‘Nam Dok Mai’	Immature	10.09 *^,ϒ^	[16]
		Mature	2.31 ^ϒ^	

^ϯ^ An asterisk (*) indicates a significant difference (*p* < 0.05) between maturity stages reported in the original cited study. ^ł^ Expressed in fresh weight (FW). ^ϒ^ Expressed in dry weight (DW).

**Table 2:** The effect of preharvest MeJa treatments on crop yield (kg tree^−1^, nº fruits tree^−1^ or plant^−1^, and fruit or mass weight) in several plant species ^ϒ^.

Fruit	Parameter	Preharvest MeJa Treatments				Reference
		Control	1 mM MeJa	5 mM MeJa	10 mM MeJa	
Pomegranate	kg tree^−1^	37.8 ± 2.1 ^c^	50.5 ± 1.8 ^a^	54.7 ± 3.5 ^a^	45.4 ± 2.8 ^b^	[18]
	Nº fruits tree^−1^	115 ± 11 ^c^	157 ± 10 ^a^	159 ± 11 ^a^	130 ± 12 ^b^	
	Fruit weight	350 ± 8 ^a^	340 ± 7 ^a^	344 ± 5 ^a^	364 ± 6 ^a^	
Pitahaya	kg tree^−1^	13.1 ± 1.2 ^c^	11.1 ± 1.1 ^d^	14.8 ± 1.2 ^b^	19.8 ± 1.3 ^a^	[19]
	Nº fruits tree^−1^	42.8 ± 2.2 ^b^	42.8 ± 2.2 ^b^	58.6 ± 3.2 ^a^	42.6 ± 2.3 ^b^	
	Fruit weight	290 ± 5 ^c^	370 ± 4 ^a^	360 ± 6 ^a^	340 ± 4 ^b^	
		**Control**	**0.1 mM MeJa**	**0.5 mM MeJa**	**1 mM MeJa**	
Lemon	kg tree^−1^	140.7± 8.5 ^a^	142.4 ± 2.8 ^a^	138.2 ± 13.5 ^a^	141.3 ± 9.3 ^a^	[20]
	Nº fruits tree^−1^	1381 ± 63 ^a^	1352 ± 105 ^a^	1381 ± 72 ^a^	1321 ± 56 ^a^	
	Fruit weight	101.8 ± 1.5 ^a^	106.3 ± 3.0 ^a^	99.7 ± 4.6 ^a^	106.9 ± 3.7 ^a^	
		**Control**		**0.5 mM MeJa**		
Artichoke	kg plant^−1^	3.19 ± 0.16		3.55 ± 0.19 *		
	Nº plant^−1^	23.12 ± 0.54		25.95 ± 0.39 *		[21]
	Mass weight	138.0 ± 1.15		144.7 ± 1.58 *		

^ϒ^ Different lowercase letters in the same row indicate statistically significant differences (*p* < 0.05) among treatments reported in the original cited study. An asterisk (*) in the same row indicates significant differences (*p* < 0.05) between treatments reported in the original cited study.

**Table 3:** Threshold safe storage temperatures and characteristic chilling injury (CI) symptoms in various fruit species. Adapted from Valero and Serrano [32] and Kader and Yahia [61].

Fruit	Threshold Safe Temperature (°C)	CI Symptoms
Banana	13–15	Peel discoloration, failure to ripen, and flesh browning.
Lemon	10–13	Pitting, surface red stains, and membrane stains.
Grapefruit	10–13	Pitting, watery breakdown, and scald.
Lime	10–13	Pitting, peel browning, and decay.
Papaya	10–12	Pitting, off-flavor, and decay (*Alternaria* spp.).
Mango	10–12	Peel discoloration, scald, off-flavor, and decay.
Pineapple	8–10	Uneven ripening, off-flavor, and wilting of crown leaves.
Pomegranate	8–10	Pitting and browning of both husk and arils.
Plum	5–7	Browning, membrane disintegration, and decay.
Orange	3–5	Pitting and brown spots on the peel.

**Table 4:** Effects of exogenous methyl jasmonate (MeJa) treatments on the physico-chemical and antioxidant properties of various fruit species.

Fruit	MeJa Concentrations	Effects on Fruit Quality Traits	Reference
Pitahaya	0.1 mM	Enhanced TSS, TA, and total phenolics.	[64]
Mango	50 µM	Delayed softening and increased TSS and color.	[76]
Eggplant	8–10 μM	Delayed the chlorophyll breakdown.	[77]
Persimmon	16 and 24 μL L^−1^	Enhanced physico-chemical characteristics.	[78]
Pomegranate	0.05 mM	Reduced browning and PPO activity.	[79]
Pear	10 μM	Increased POD, CAT, and SOD activities.	[75]
Banana	100 μM	Reduced weight losses and softening.	[80]
Pomegranate	0.01 mM	Increased color and total anthocyanins.	[81]
Plum	25 ppm	Improved firmness, TSS, and TA.	[82]
Peach	1 mM	Delayed softening and browning.	[83]
Pomegranate	0.01 and 0.1 mM	Enhanced total antioxidant activity.	[84]
Blood orange	40 mM	Increased the activity of antioxidant enzymes.	[85]
Jujube	0.2 and 0.4 mM	Reduced weight losses and increased TSS and TA.	[86]
Pomegranate	5 mM	Enhanced total phenolics and anthocyanins.	[87]

## Data Availability

No new data were created or analyzed in this study.
